# The Study of Cooperative Obstacle Avoidance Method for MWSN Based on Flocking Control

**DOI:** 10.1155/2014/614346

**Published:** 2014-02-10

**Authors:** Zuo Chen, Lei Ding, Kai Chen, Renfa Li

**Affiliations:** ^1^College of Information Science and Engineering, Hunan University, Changsha, Hunan 410082, China; ^2^College of Information Science and Engineering, Jishou University, Jishou, Hunan 416000, China

## Abstract

Compared with the space fixed feature of traditional wireless sensor network (WSN), mobile WSN has better robustness and adaptability in unknown environment, so that it is always applied in the research of target tracking. In order to reach the target, the nodes group should find a self-adaptive method to avoid the obstacles together in their moving directions. Previous methods, which were based on flocking control model, realized the strategy of obstacle avoidance by means of potential field. However, these may sometimes lead the nodes group to fall into a restricted area like a trap and never get out of it. Based on traditional flocking control model, this paper introduced a new cooperative obstacle avoidance model combined with improved SA obstacle avoidance algorithm. It defined the tangent line of the intersection of node's velocity line and the edge of obstacle as the steering direction. Furthermore, the cooperative obstacle avoidance model was also improved in avoiding complex obstacles. When nodes group encounters mobile obstacles, nodes will predict movement path based on the spatial location and velocity of obstacle. And when nodes group enters concave obstacles, nodes will temporarily ignore the gravity of the target and search path along the edge of the concave obstacles. Simulation results showed that cooperative obstacle avoidance model has significant improvement on average speed and time efficiency in avoiding obstacle compared with the traditional flocking control model. It is more suitable for obstacle avoidance in complex environment.

## 1. Introduction

Traditional mobile wireless sensor mainly uses space fixed sensor nodes for data collection. Although these nodes' costs are rather low, due to the limitation of communication and detection range, this fixed network topology cannot satisfy the micro, mobile application requirement in the future. Mobile wireless sensor network (MWSN) [1] has better adaptability and robustness for the changes of the environment, and the realization of all kinds of new models becomes a possibility. Construction of mobile wireless sensor network by adding some mobile nodes has become a hot spot in current research field. Based on the feature of mobility of sensor nodes in MWSN, this paper introduces this feature into target tracking and has wide application in reality, for instance, tracking wildlife habits, detecting patients' health status, and disaster rescuing. In particular, MWSN showed great advantage in target tracking in bad conditions.

Unlike space fixed WSN, the network constructed by mobile wireless sensors is more like a swarm system and the whole group present flocking behavior [2] through individual local information. There is a problem in target tracking by using mobile sensors: with limited computing resources and detecting range [3], how to make sure sensor nodes do not collide during tracking the target, and sensor nodes can cooperatively choose their path when group encounters obstacles. Flocking control method [2] is one of the most popular cooperative control methods. Flocking can be defined as follows: a group of independent agents keep the form of a team moving towards a certain destination, and it is a team behavior. Lots of mobile sensor nodes communicate with each other and make sure there are no collision, matching velocity, and gathering towards the group centre. Therefore, according to the features of swarm system, it is necessary to build a cooperative control model for mobile wireless sensor nodes in target tracking. 

In the meantime, in practical application of mobile wireless sensor target tracking, the operation environment of nodes is rather complicated. There exist not only mobile obstacle, but also concave obstacles. Due to the relative speed between nodes and obstacles, it is likely for collision when nodes encounter mobile obstacles to occur. Besides, concave obstacle with various kinds of holes can easily trap the sensor nodes and lead to the failure of target tracking. This bad situation affects the efficiency of target tracking [4], even gradually away from the destination. 

Aiming at above problems, this paper studied the target detecting [5] methods in mobile wireless sensor network, improved the flocking control model in swarm system, realized and improved SA obstacle avoidance algorithm, and introduced an efficient cooperative obstacle avoidance model. The system constructed by nodes has wider detecting range than individual node, and it will make more reasonable judgment during the target tracking or obstacle avoidance. This paper further improved the obstacle avoidance algorithm, in order to make up its defect in complex environment, this paper further improved the obstacle avoidance algorithm. Group will predict the steering path when it encounters mobile obstacles. While group encountered concave obstacles, after group entered the concave obstacle, group will judge its own environment, searching the path along the edge of obstacle, and will finally exit the concave obstacle and continue to track the target. Simulation results showed that, compared with traditional flocking control model, cooperative obstacle avoidance model has significantly improved in average speed and time efficiency in avoiding obstacle, and it is applied to avoid the complex obstacles.

## 2. Related Researches

In the research of target tracking of MWSN, an important problem is how to make sure nodes do not collide and separate during target tracking, and nodes' velocity stays in a reasonable state. Flocking control model has always been a hotspot in this research field.

In 1987, Reynolds introduced a model [6] to simulate swarm behavior by using computer in three dimensions. This model includes the following rules: (1) separation: avoid collision with other nodes in detecting range; (2) coherence: stay close to other nodes in detecting range; (3) matching velocity: match other nodes average velocity in detecting range. Reynolds specifically studied the flocking behavior in biosphere, and although Reynolds did not give a concrete model, these three rules lead to a new direction in the research of flocking control.

Tanner et al. [7–9] were inspired by Reynolds' model, they introduced a swarm system with double integral feature and realized the above three rules in Reynolds model. In article [7], Tanner et al. mainly concerned the fixed network topology; individual agent only needs to communicate with a few particular agents. In article [8], Tanner et al. introduced their model into dynamic topology; agent's neighborhood will change with the time; in other words, only agents within the neighborhood area can communicate with each other. Individual's control input is determined by the other agents with its detecting range. Article [9] introduced flocking control model into multiple static obstacles environment; group steers its direction to avoid obstacles during the target tracking procedure. Group might separate into several subgroups to avoid obstacles and gather together to continue to track the target. However, due to the limits of the model itself, efficiency of obstacle avoidance is rather low, and parts of agent cannot reach the destination. Articles [10, 11] further studied Tanner's model and introduced the concept of virtual leader. It concerned virtual leader as a new control input. Average velocity of group will stay the same as the virtual leader. Virtual leader can avoid the separation of group. In Tanner's model, agent's control input is separated into several parts and different control is used to accomplish different goals. If there comes a new demand, model only needs to add new control input. This model can adjust different control inputs to adapt to new environment.

Flocking control model accomplishes the goal of target tracking; it fits the requirement of target tracking in MWSN. Tanner's model is much simpler than others. Simulation results showed that this model has good cooperative control ability too. Besides, flocking control model joined obstacle avoidance algorithm. However, efficiency of its obstacle avoidance algorithm is rather low. It exerts a repulsive force when agent encounters obstacles. This may cause unnecessary waste in agent's velocity and motion path during the obstacle avoidance procedure. Aiming at this problem, we need to introduce an efficient obstacle avoidance algorithm into Tanner's flocking control model.

Although flocking control model accomplished the target tracking goal in MWSN, group needs to avoid obstacles during the tracking procedure. Its obstacle avoidance algorithm directly determined the motion path of group. Virtual force obstacle avoidance algorithm [12, 13] is main subject in the current. It exerts a repulsive force on agent when agent encounters obstacles; agent cannot get near to the obstacle. Virtual force model is very convenient to establish, but it only slows down agent's velocity during the obstacle avoidance procedure. Without steering judgment, efficiency of obstacle avoidance is rather low.

Potential field method is a common method in robot path planning. This method introduced a virtual potential field to control the motion of robot. Target produces gravitational potential field, and obstacle produces repulsion potential field. With those two potential fields, if agents are moving towards the negative gradient direction of the potential field, group might be trapped into concave obstacle. And group cannot reach the destination due to the local minimum point. Aiming at the above problems, scholars did many beneficial attempts. Article [14] introduced local minimum recover method; combined with potential field method and virtual force algorithm, it overcomes the local minimum problem in concave obstacle environment. Due to lack of research efforts, obstacles are heuristic, and the algorithm efficiency is only based on simulation results. Article [15] introduced improved artificial potential field method, combined with potential field method and genetic algorithm (GA), but it needs too much computer resources and does not apply to real-time application. Besides, some articles introduced best-first search [15], simulated annealing algorithm [16], and immediately search [17, 18] into potential field method and search for a lower potential field value than the local minimum point and move along with the negative gradient direction till the group reaches the destination. Without enough heuristic information, efficiency of this search method is very low. Thus, it always comes to a failure in concave obstacle environment.

In Reynolds' article, he introduced an obstacle avoidance algorithm, Steer to Avoid (SA) algorithm. Some scholars realized similar algorithm [19]. SA algorithm combined with biological characteristics, we could regard this algorithm as the birds' navigation in nature. This obstacle algorithm has the following features: agent is only concerned with the obstacle right in front of its heading direction, operates in its own coordinate system, and is concerned with the right angle direction of the obstacle's centre and itself as steering direction. SA algorithm allows agents moving along with the edge of obstacle; it has better understanding of the obstacle information and further improved obstacle avoidance efficiency.

In conclusion, it is of great importance to avoid obstacles with high efficiency in flocking control model. SA algorithm is an efficient obstacle avoidance algorithm, but it has its own shortage. We need to establish a cooperative obstacle avoidance model by joining efficient obstacle avoidance algorithm in flocking control model. Cooperative obstacle avoidance model can avoid obstacles with high efficiency during the target tracking procedure.

## 3. Cooperative Obstacle Avoidance Model

### 3.1. Problem Description

**Figure 1:**
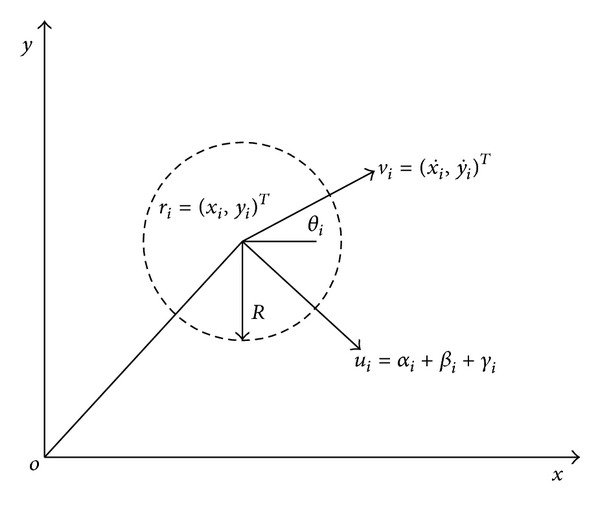
Cooperative obstacle avoidance model.

There are *N* agents on a plate. Agent size will be ignored, and its mass is 1. Agents are equipped with sensors, so we can regard every agent as a sensor node. Sensors can receive information from its neighborhood agent, such as position and velocity. We assume that the detection angle is 2*π*. Agents are moving with the following functions:

(1) $$\begin{array}{c} {\dot{r}}_{i}=v_{i},\\ {\dot{v}}_{i}=u_{i},\;i=1,\ldots ,N, \end{array}$$

where *r*_*i*_ = (*x*_*i*_,*y*_*i*_)^*T*^ is the position of agent *i*; $v_{i}={\left({\dot{x}}_{i}+{\dot{y}}_{i}\right)}^{T}$ is its velocity; the included angle between*v*_*i*_ and horizontal direction will be *θ*_*i*_, $\tan q_{i}={\dot{x}}_{i}/{\dot{y}}_{i}$, and $u_{i}=({\ddot{x}}_{i}+{\ddot{y}}_{i})$ is its control input. Relative position vectors are denoted *R*_*ij*_ = *r*_*i*_ − *r*_*j*_. The control input consists of three components (Figure 1):

(2) $$\begin{array}{c} u_{i}=\alpha_{i}+\beta_{i}+\gamma_{i}. \end{array}$$

The first component, *α*_*i*_, is the velocity matching term; it implements the third goal in Reynolds' model. *β*_*i*_ is the synergy term and it implements the first two goals in Reynolds' model. *γ*_*i*_ is the virtual leader term; it can produce a repulsive force which let the agent *i* move away fromaway from obstacles and leads agents moving towards destination *r*_*d*_ = (*x*_*d*_,*y*_*d*_)^*T*^.

### 3.2. Model Establishment

Neighborhood (detection zone), because of sensor node, can only communicate with nodes in its detection range, and the control input is acquired from this information mostly. *N*_*i*_ is a node set that indicates which node inside the detection zone of node *i*:

(3) $$\begin{array}{c} N_{i}=\left\{||r_{ij}\leq R||\right\}\subseteq \left\{1,\ldots ,N\right\}, \end{array}$$

where *R* is the detection radius of node *i*. Because nodes are moving all the time, the distance between nodes is not constant, so the neighborhood agents will change with time.

Velocity matching control input *α*_*i*_: goal of this control input is to keep node's velocity equal to the neighbor's:

(4) $$\begin{array}{c} \alpha_{i}=-\sum_{j\in N_{i}}\left(v_{i}-v_{j}\right). \end{array}$$

Synergy control input *β*_*i*_: this control input exerts a repulsion force while two nodes stay too close and exert an attraction force if the distance between two nodes is too far. When the distance is longer than *R*, there is no force at all. The combined effects of node *i*'s neighboring nodes construct the control input *β*_*i*_. *V*_*ij*_ is the control input to node *i*, which is exerted by node *j*:

(5) $$\begin{array}{c} V_{ij}={\left(r_{ij}-r\right)}^{n};\;n=1,3,5,\ldots ,2k+1. \end{array}$$

As the group is stabilized, the distance between two nodes will be *r*. *n* is an odd number of positive integer which depends on the situation. We can define the synergy control input *β*_*i*_ as

(6) $$\begin{array}{c} \beta_{i}=-\sum_{j=1,i\neq j}V_{ij}. \end{array}$$

Virtual leader control input *γ*_*i*_: the mission of this term is to lead nodes moving towards the destination *r*_*d*_ and decelerates when nodes encountered an obstacle. When the obstacle is out of node's detection range, this control input exerts a constant force *U*_*i*_ = *U*_*d*_ on node towards destination, and it can lead node moving towards destination. When node's velocity reaches a maximum value *v*_max⁡_, it cancels the exertion of *U*_*i*_. When node is close to the destination *r*_*id*_ < *r*_min⁡_, cancel this force too. And let node finally stay around the destination. When node detects an obstacle, we first cancel this attraction force, and seek the nearest point *r*_*o*_ on obstacle, exerting a repulsion force on node *i*:

(7) $$\begin{array}{c} U_{i}=\frac{-U_{o}}{{\left(r_{io}\right)}^{m}}, \end{array}$$

where *U*_*o*_ is a constant value, and *m* is a positive integer; we can define the virtual leader control input as *γ*_*i*_ = *U*_*i*_.

Thus, the total control input *u*_*i*_ of node *i* can be defined as

(3.2) $$\begin{array}{c} u_{i}=\{\begin{array}{cc} -\sum_{j\in N_{i}}\left(v_{i}-v_{j}\right)-\sum_{j\in N_{i}}V_{ij}-U_{i}-kv_{i}; & r_{id}<r_{\min}\\ -\sum_{j\in N_{i}}\left(v_{i}-v_{j}\right)-\sum_{j\in N_{i}}V_{ij}-U_{i}; & \text{otherwise}. \end{array} \end{array}$$

When nodes are close to the destination *r*_*id*_ < *r*_min⁡_, exert a damping control −*kv*_*i*_ on nodes.

### 3.3. SA Algorithm

Tanner's model realized the three flocking laws of Reynolds' model. However, without any steering judgment, this model only slows nodes down while it encounters the obstacle. We need to add an efficient obstacle avoidance algorithm. Here, we introduced SA algorithm.

Traditional SA algorithm only concerns the obstacles right in front of node's velocity direction. This approach exerts a steering control input on node. The direction of this control input is in vertical direction of the line which links the center of the obstacle and the intersection point on obstacle of velocity line and the obstacle edge. However, in reality, the center of the obstacle is difficult to determine, and sensors do not have such a long detection range. So, this paper improved Steer to Avoid obstacle avoidance algorithm, defining turning direction as the tangent line of the intersection point on obstacle of velocity line and the obstacle edge. It leads nodes group to avoid the obstacle efficiently.

Procedures of the SA obstacle avoidance algorithm can be described as follows.

**Step 1**

When sensor detects an obstacle, calculate the tangent line of the intersection point on obstacle of velocity line and the obstacle edge.

**Step 2**

Tangent line has two directions. We choose one direction randomly; this azimuth is recorded as *σ*_*i*_ (all the azimuth is relative to the horizontal). Then, we connect the node *i* and destination *r*_*d*_: the azimuth of this line is recorded as *ξ*_*i*_, and this line is bound to intersect with the tangent line:

(9) $$\begin{array}{c} \xi_{i}=\arctan\left(\frac{\left(y_{i}-y_{d}\right)}{\left(x_{i}-x_{d}\right)}\right). \end{array}$$

**Step 3**

**Figure 2:**
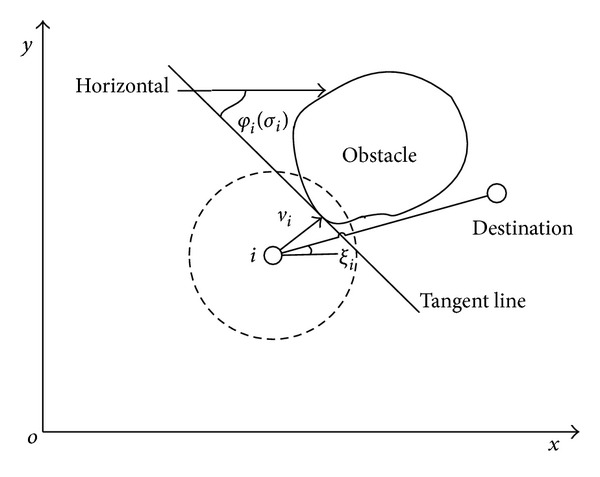
Direction judgment in SA algorithm.

Based on Step 2, if we choose *σ*_*i*_ as the turning azimuth, after node turned its direction, we project velocity on *ξ*_*i*_ line, and we can attain a velocity component. Then, use *σ*_*i*_ subtract *ξ*_*i*_, if result is an acute angle, it means the velocity component points are at *r*_*d*_, so the group is moving towards *r*_*d*_. We choose *σ*_*i*_ as the turning azimuth. Reversely, if the result is an obtuse angle, we use *σ*_*i*_ subtract *π*. Finally, we can attain a turning azimuth *ϕ*_*i*_. It is shown in Figure 2 *φ*_*i*_ can be described as

(10) $$\begin{array}{c} \varphi_{i}=\{\begin{array}{cc} \sigma_{i}; & \left|\sigma_{i}-\xi_{i}\right|\leq \frac{\pi }{2}\\ \sigma_{i}-\pi ; & \text{otherwise.} \end{array} \end{array}$$

In addition, it is possible that some nodes do not detect the obstacle (the turning azimuth is null), but its neighboring nodes have changed its direction (the turning azimuth is not null). These nodes can make a turning decision according to the information from its neighborhood. This decision is made before nodes detect the obstacle, so the efficiency of obstacle avoidance is improved.

Before a node makes a turning decision, firstly, we check if this node detected an obstacle. Node chooses a turning direction according to the SA algorithm. If there was no obstacle, node will examine all the neighboring nodes' turning azimuth, and attain an average turning azimuth. There are *n* nodes in the neighborhood nodes of node *i*, in which their turning azimuth is not null. It can be described as

(11) $$\begin{array}{c} \varphi_{i}=\sum_{j\in N_{i}}\frac{\varphi_{j}}{n};\;j\in N_{i},\;\;\varphi_{j}\neq \text{null}. \end{array}$$

In conclusion, through the above model, it leads nodes group moving towards the destination cooperatively and avoids the obstacle with a high efficiency. What more, a single node's detection range is rather small, because they are lots of nodes in a group; these nodes construct a huge detection zone. This will let nodes group make its steering decision more wisely.

### 3.4. Cooperative Obstacle Model in Complex Environment

If there existed mobile obstacle in environment, current model's steering judgment might be the same as the velocity direction of obstacle. It leads to the decrease of obstacle avoidance efficiency. The following two solutions based on the features of mobile obstacle combined with SA algorithm in the obstacle avoidance procedure.

#### 3.4.1. Movement Prediction

According to the mobile obstacle's velocity, nodes could predict the position of obstacle after Δ*t* seconds (assume that obstacle is moving with the same speed). Nodes apply its SA algorithm based on the predicted position. Thus, node group improved its obstacle avoidance efficiency according to its moving tendency. While obstacles entered a node's detecting range, it calculates mobile obstacle's instant velocity *v*_*o*_ through the differential of obstacle's position. We could decompose *v*_*o*_ into vertical and horizontal direction:

(12) $$\begin{array}{c} v_{ox}=v_{o}\times \cos\theta_{o},\\ v_{oy}=v_{o}\times \sin\theta_{o}. \end{array}$$

*θ*_*o*_ is the included angile between the obstacle velocity direction and horizontal direction. Position of points (*O*_*x*_, *O*_*y*_) on the obstacle after Δ*t* seconds will be

(13) $$\begin{array}{c} O_{x}=O_{x}+v_{ox}\times \Delta t,\\ O_{y}=O_{y}+v_{oy}\times \Delta t. \end{array}$$

**Figure 3:**
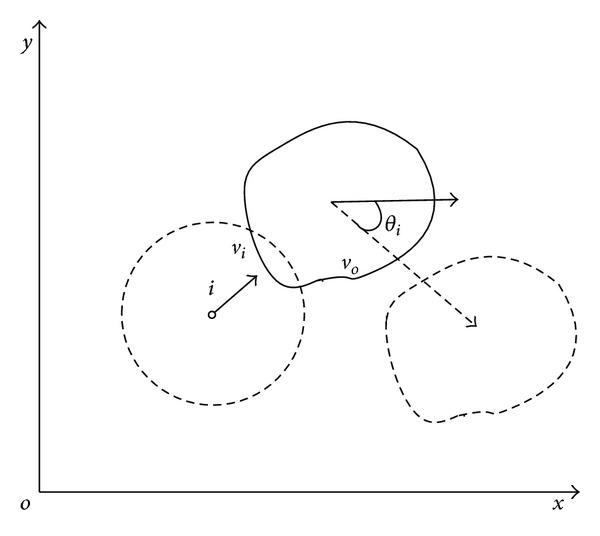
Movement Prediction.

As shown in Figure 3, node applies its SA algorithm according to the obstacle's position after Δ*t* seconds. Obstacle is already not on the node's velocity direction. And node will not change its direction. In reality, Δ*t* is rather short.

#### 3.4.2. Obstacle Velocity

With the composed effects of the above model, it only leads nodes moving along with the edge of obstacle. However, if obstacle tends to move away from nodes, efficiency of obstacle avoidance will decrease. If obstacle tends to move towards nodes, SA algorithm cannot adjust node's velocity into a reasonable value. Thus, velocity of obstacle should be considered in the SA algorithm.

**Figure 4:**
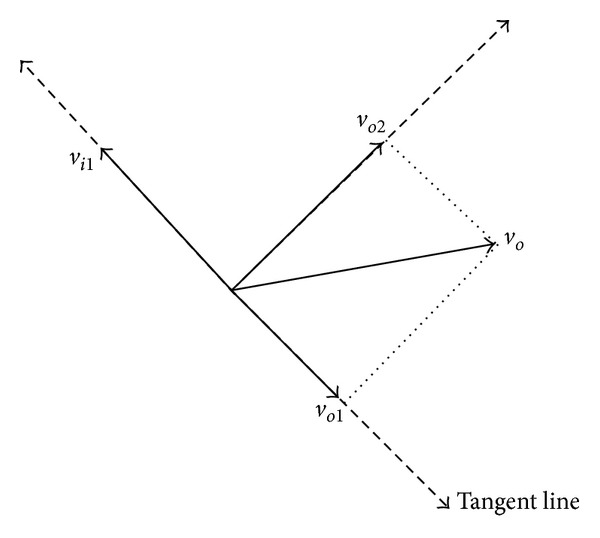
Projection of obstacle velocity.

According to the original SA algorithm, during the obstacle avoidance procedure, one tangent direction will be selected as the node's steering direction. As shown in Figure 4, we assume that *v*_*i*1_ is the node's velocity after the SA algorithm. We decompose obstacle's velocity by projecting it into node's SA coordination. This coordination uses the tangent direction as the *X*-axis and the vertical direction of the tangent line as the *Y*-axis. It attains two velocity components, *v*_*o*1_ and *v*_*o*2_.

We can project obstacle's velocity into node's SA coordination by the following simultaneous equations from (14) to (18):

(14) $$\begin{array}{c} v_{o1x}+v_{o2x}=v_{ox}, \end{array}$$

(15) $$\begin{array}{c} v_{o1y}+v_{o2y}=v_{oy}, \end{array}$$

(16) $$\begin{array}{c} v_{o1x}^{2}+v_{o2x}^{2}+v_{o1y}^{2}+v_{o2y}^{2}=v_{ox}^{2}+v_{oy}^{2}, \end{array}$$

(17) $$\begin{array}{c} \tan\varphi_{i}=\frac{v_{o1y}}{v_{o1x}}, \end{array}$$

(18) $$\begin{array}{c} \tan\left(\varphi_{i}+\frac{\pi }{2}\right)=\frac{v_{o2y}}{v_{o2x}}. \end{array}$$

If *v*_*o*1_ and *v*_*i*1_ are on the opposite direction, it is suitable for nodes group to avoid obstacle, so we choose *v*_*i*1_ as the final steering direction. If *v*_*o*1_ and *v*_*i*1_ are on the same direction, it means that nodes group tends to move with the same direction of obstacle. In this situation, if the speed of obstacle is rather slow, nodes group could accelerate to go around obstacle, and if obstacle's speed is close to node's speed, we need to reverse *v*_*i*1_. If *v*_*o*1_ is smaller than half of *v*_*i*1_, select *v*_*i*1_ as the steering velocity, and if *v*_*o*1_ is larger than half of *v*_*i*1_, reverse *v*_*i*1_ as the steering direction. We could define *v*_*i*1_ in the following:

(19) $$\begin{array}{c} v_{i1}=\{\begin{array}{cc} v_{i1};v_{i1},v_{o1}\;\;\text{opposite}, & \text{or}\;\;v_{o1}<\frac{1}{2}v_{i1},\\ -v_{i1};v_{i1},v_{o1}\;\;\text{same}, & \text{and}\;\;v_{o1}>\frac{1}{2}v_{i1}. \end{array} \end{array}$$

Combine *v*_*i*1_ and *v*_*o*1_, and we could obtain the final steering velocity *v*′_i_. Because this steering velocity was determined by the tendency of obstacle's movement, the efficiency of obstacle avoidance is much more higher:

(20) $$\begin{array}{c} v_{ix}^{\prime }=v_{o2x}+v_{i1x},\\ v_{iy}^{\prime }=v_{o2y}+v_{i1y}. \end{array}$$

In reality, the environment of nodes group is very complicated, not only in mobile obstacles, but also in some concave obstacles. While nodes group encountered concave obstacles, due to the limitation of nodes' detecting range, nodes group cannot determine its tendency of entering the concave obstacle. According to the above model, after nodes detect the concave obstacle, nodes will use SA algorithm to avoid the obstacles. In the concave obstacle, with gravitation of the destination, nodes group will be trapped in the obstacle.

Local information cannot let nodes make a reasonable path choice due to the limitation of the detecting range. No matter whether concave obstacles or convex obstacles, first time of the SA obstacle avoidance judgment is always the same. Because the gravitation force of destination might affect the path search procedure, we cancel this gravitation force after nodes detected obstacles during the SA obstacle avoidance procedure. In order to make sure nodes group do not move away from the obstacle, here we introduced a gravitation force to the obstacles:

(21) $$\begin{array}{c} U_{i}^{\prime }=\{\begin{array}{cc} U_{o}^{\prime }\;r_{io}; & 0<r_{io}\leq R,\\ 0; & r_{io}>R, \end{array} \end{array}$$

where *U*_*o*_′ is a constant value and *r*_*io*_ is the closest point on the obstacle *O* to the node. This gravitation force applies to node's obstacle avoidance procedure, combined with effect of (7). It makes sure nodes moving along with edge of the obstacle and keeps obstacle in node's detecting range.

After the SA obstacle avoidance judgment, we need to let nodes group move along with the edge of obstacle, without the effect of the target's gravitation. In the SA algorithm, we could attain two tangent angels: *σ*_*i*_ and *σ*_*i*_ − *π*; we need to choose one tangent angel as the steering direction. According to the local information in the node's detecting range, we could choose the steering direction based on the following three situations.

**Figure 5:**
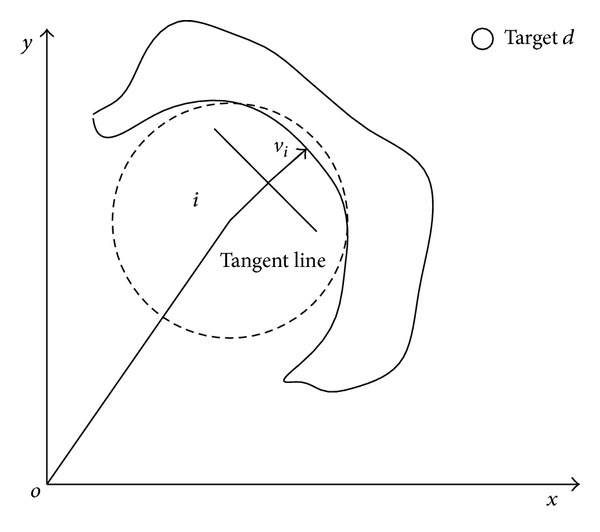
No obstacle was detected on both two directions.

**Figure 6:**
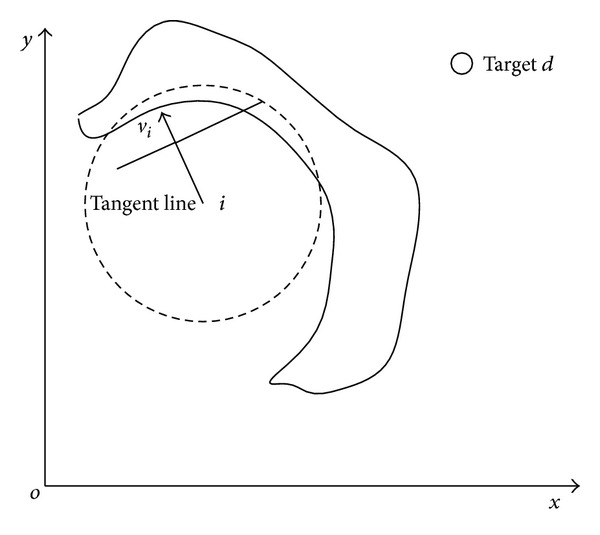
Obstacle was detected in one direction.

**Figure 7:**
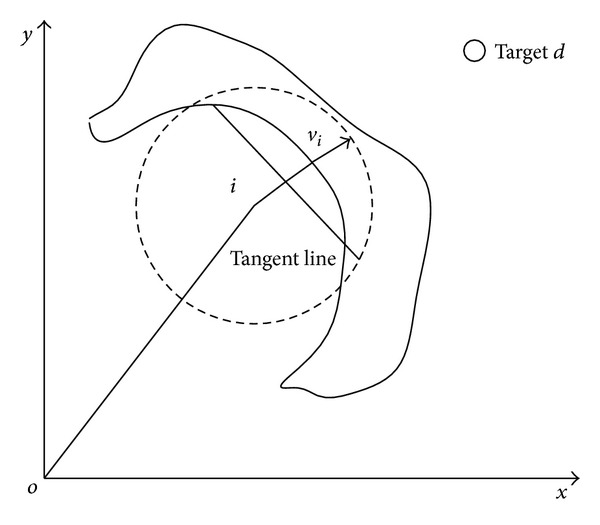
Obstacles were detected in both two directions.

(1) No obstacle was detected on both two tangent directions. Due to the limitation of the detecting range, and the tangent line is on the edge of obstacle, it is hard for the nodes to attain local information. Here, we move tangent line to the centre of node *i* and tangent point *o* and determine if this line intersects with obstacles. As shown in Figure 5, because node cannot determine if it has already entered the concave obstacle, it uses the original SA algorithm in (10) to avoid obstacle. Based on (22), we could get the tangent line *l*, where (*x*_*o*_, *y*_*o*_) is the tangent point of the obstacle and node's velocity line:
  (22) $$\begin{array}{c} Y-\frac{\left(y_{i}-y_{o}\right)}{2}=\sigma_{i}\left(X-\frac{\left(x_{i}-x_{o}\right)}{2}\right). \end{array}$$
(2) Obstacle was detected on one tangent direction. As shown in Figure 6, node could determine that it is in the concave obstacle. In order to let node searching path along with the edge of obstacle, here we ignored the SA judgment which is close to the target. Tangent line only got one intersection with the edge of obstacle in node's detecting range. Select the tangent direction which is without obstacle in the detecting range.

(3) Obstacles were detected on both two tangent directions. As shown in Figure 7, node cannot exit concave obstacle if nodes choose tangent direction as steering direction. Tangent line has two intersections with the edge of obstacle. In order to make sure node could exit the obstacle, we reverse node's velocity direction as the steering direction.

The final steering angel *φ*_*i*_ can be described by the following, where *θ*_*i*_ is node's velocity angel before the SA algorithm.

(23) $$\begin{array}{c} \varphi_{i}=\{\begin{array}{cc} \text{Equation}\;\left(10\right); & \text{no}\;\;\text{obstacle}\;\;\text{was}\;\;\text{detected},\\ \sigma_{i}; & \text{obstacle}\;\;\text{was}\;\;\text{detected}\;\;\text{only}\;\;\\ & \text{in}\;\;\text{direction}\;\;\sigma_{i},\\ \sigma_{i}-\pi ; & \text{obstacle}\;\;\text{was}\;\;\text{detected}\;\;\text{only}\;\;\\ & \text{in}\;\;\text{direction}\;\;\sigma_{i}-\pi ,\\ \theta_{i}-\pi ; & \text{obstacle}\;\;\text{was}\;\;\text{detected}\;\;\text{in}\;\\ & \text{both}\;\;\text{two}\;\;\text{direction}. \end{array} \end{array}$$

With the above equation, nodes search the exit path along with the edge of obstacle by applying improved SA algorithm. And the gravitation and the repulsion forces keep nodes moving along with the edge of obstacle. In order to make sure nodes do not enter the concave obstacle again, here we record *U*_*i*_′ before nodes exit the obstacle and exert this force for another Δ*t* seconds. Δ*t* is dependant on nodes' velocity, and it can be defined by the following, where *T* is a constant value. After Δ*t* seconds, resume the gravitation force of the target,

(24) $$\begin{array}{c} \Delta t_{i}=\frac{T}{v_{i}}. \end{array}$$

Through the above model, mobile wireless sensor nodes constructed a stable group and move towards the target. During the target tracking procedure, nodes group might encounter some obstacles. Model cancels the gravitation force of the target and lets nodes group searching path along with the edge of obstacle. It increases the efficiency of obstacle avoidance. While nodes group encountered mobile obstacle, nodes will predict the obstacle's position according to the velocity of obstacle. While nodes group encountered concave obstacles, nodes could determine that they have already entered the concave obstacle. Nodes could adjust its velocity into a reasonable direction, search for the path along with the edge of obstacle, and finally avoid the concave obstacle.

## 4. Simulation and Analysis 

Section 3 has already realized cooperative obstacle avoidance model. Experiments simulate the cooperative obstacle avoidance model and prove the effectiveness and stability of the model. Because the model was based on Tanner's flocking control model, simulation will compare the model with flocking control. Simulation records the velocity and position of 10 sensor nodes and analysis will be made based on the simulation result.

Initiate the 10 sensor nodes as follows: (1) initial velocity is a random value between (−100, −100) and (100, 100). (2) Detecting range of the sensor nodes is 150. (3) Initial control input is 0. (4) Sensor nodes initiate the same coordination system, but only operate in its own coordination.

After the initialization, these sensor nodes move towards the target cooperatively and avoid the complex obstacles in the environment. Simulation records the motion path of 10 sensor nodes. Horizontal axis is the *x* position of nodes, and vertical axis is the *y* position of nodes.

In simulation, mobile wireless sensor nodes move as a group. There is no collision or separation during the target tracking procedure. Single node's velocity is always close to the average velocity of the group. Nodes group moves towards the target with the gravitation force of the target.

### 4.1. Simulation in Static Obstacle Environment

**Figure 8:**
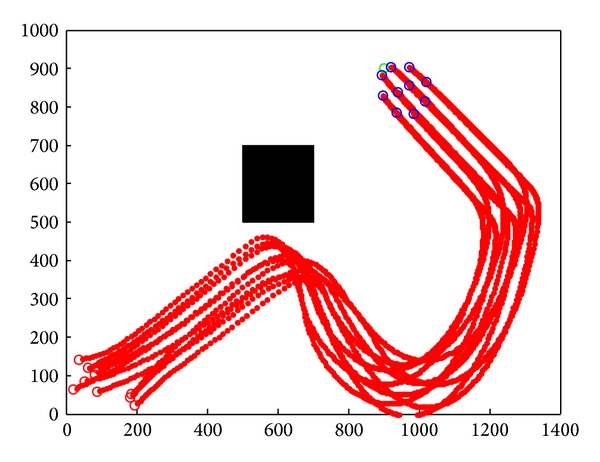
Static obstacle avoidance by applying flocking control model.

Simulation result is shown in Figure 8 by applying Tanner's flocking model. While nodes group encountered static obstacles, with the effects of the control input, nodes group only stay far from the obstacle and move towards the target again. Due to the huge cost of nodes' speed, nodes reached target after two deceleration.

**Figure 9:**
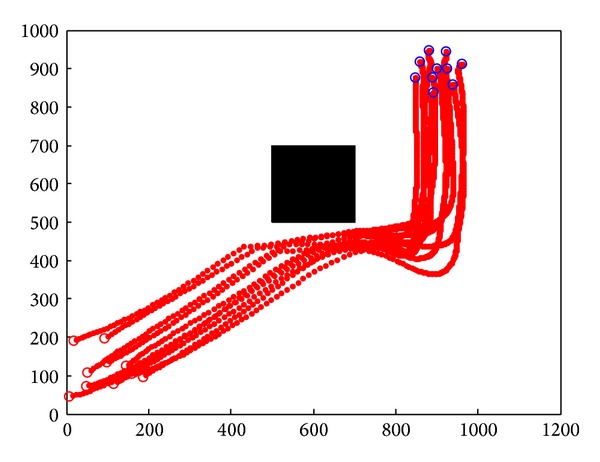
Static obstacle avoidance by applying cooperative obstacle avoidance model.

In Figure 9, we applied the cooperative obstacle avoidance model with SA algorithm. While sensor nodes encountered static obstacles, nodes could make the obstacle avoidance judgment instantly and move along with the edge of the obstacle. Nodes which encountered obstacle first could inform the nodes that did not. Simulation results showed that cooperative obstacle avoidance model improves the efficiency of obstacle avoidance.

**Figure 10:**
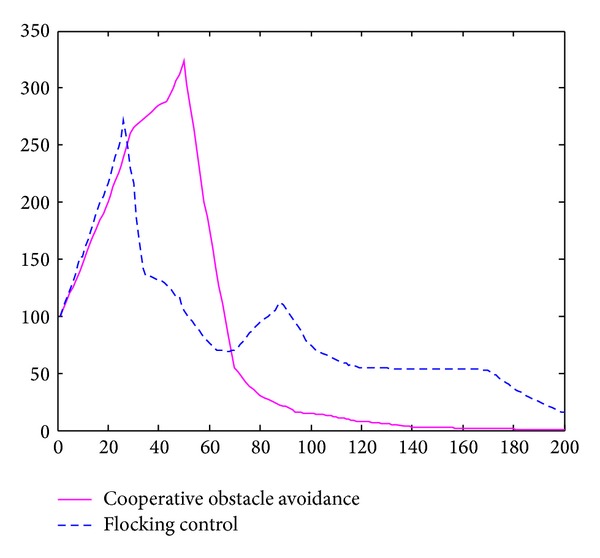
Speed curve of static obstacle avoidance.

Based on the simulation results, the average motion path length and the average speed of sensor nodes could also be regarded as an important value to determine efficiency of the model. In Figure 10, cooperative obstacle avoidance model moved less distance than the flocking control model. It chose better motion path, with less deceleration.

In Figure 10, horizontal axis is the movement time of the nodes group. Vertical axis is the average speed of the 10 nodes. The blue curve is the application of Tanner's flocking control model. The crimson curve is the application of cooperative obstacle avoidance model. We could draw a conclusion based on this simulation result. Flocking control model had a huge deceleration when nodes encountered obstacle. It needs 200 seconds to reach the destination approximately. Cooperative obstacle avoidance model only had a small deceleration during the obstacle avoidance; It regains the velocity soon after the SA steering judgment. Simulation result showed that cooperative obstacle avoidance model only needs 80 seconds to reach the target. Thus, cooperative obstacle avoidance model could avoid the obstacle with a higher speed and efficiency and reach the target much more sooner.

### 4.2. Simulation in Mobile Obstacle Environment

**Figure 11:**
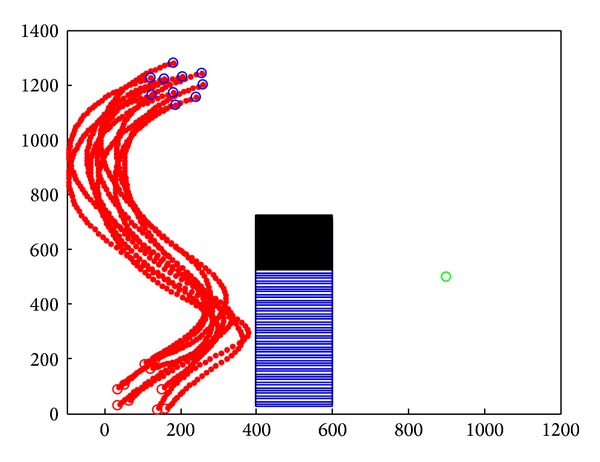
Mobile obstacle avoidance by applying flocking control model.

Nodes group avoids the mobile obstacle by applying flocking control model; the simulation result is shown in Figure 11.

Tanner's flocking control model only exerts a repulsion force on nodes, without any steering judgment. As a result of that, if nodes decrease their speed when encounter obstacle, it will take a long time for them to regain the velocity. During this procedure, obstacle is also moving, and nodes group might encounter the obstacle again. Thus, efficiency of flocking control model applied in mobile obstacle environment is rather low.

**Figure 12:**
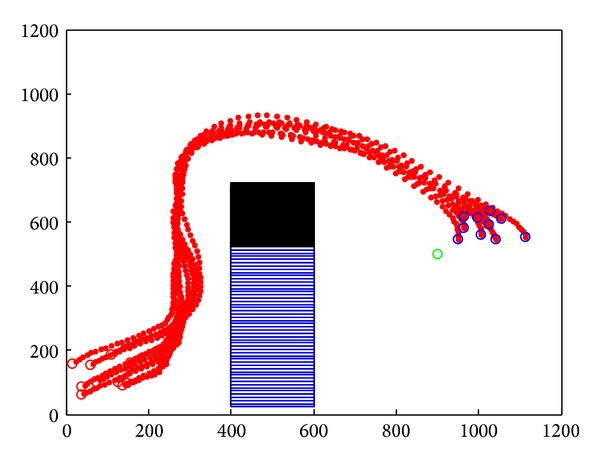
Mobile obstacle avoidance by applying original cooperative obstacle avoidance model.

Simulation result of original cooperative obstacle avoidance model applied in mobile obstacle environment is shown in Figure 12. With the SA steering judgment, the steering direction was chosen as the tangent direction. The efficiency of obstacle avoidance is higher than the flocking control model. But in the mobile obstacle environment, steering judgment of SA algorithm might not be the best. If the steer direction is the same as the mobile obstacle's velocity direction, nodes group and obstacle remained relatively static. The efficiency of obstacle avoidance decreased.

**Figure 13:**
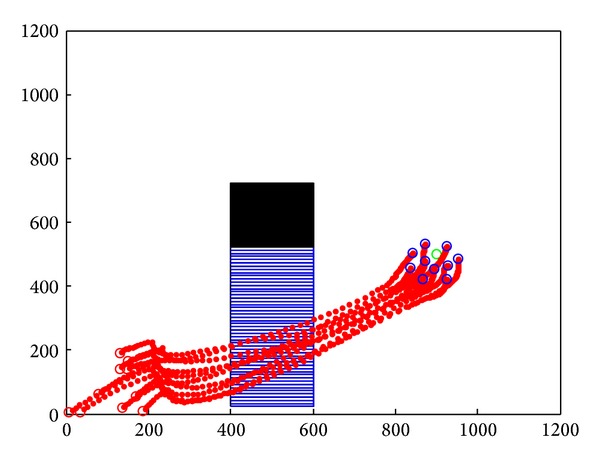
Mobile obstacle avoidance by applying improved cooperative obstacle avoidance model.

Improved cooperative obstacle avoidance model was applied in the mobile obstacle environment; it combined the effects of obstacle's position and velocity. Simulation result is shown in Figure 13. If the SA steering velocity is close to obstacle's velocity, nodes could reverse its velocity direction. What is more, nodes predict the mobile obstacle's position before the SA algorithm. Improved obstacle avoidance model increased the efficiency of obstacle avoidance.

**Figure 14:**
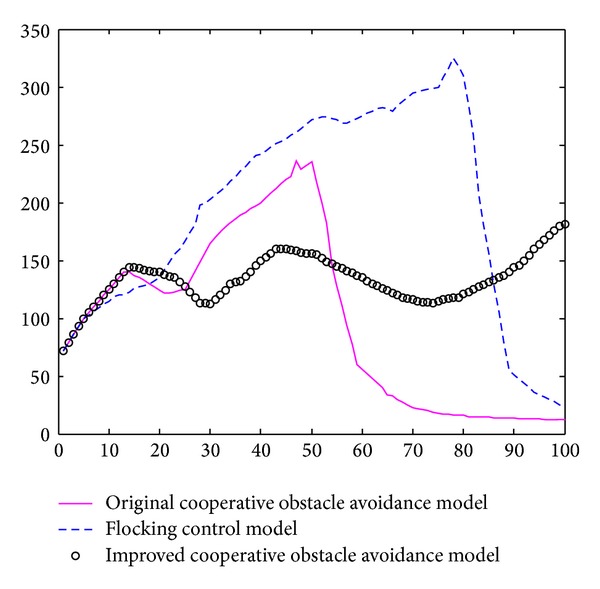
Speed curve of mobile obstacle avoidance.

Figure 14 revealed the speed curves of the above three different models.

In Figure 10, horizontal axis is movement time of the nodes group. Vertical axis is the average speed of the 10 nodes. The blue curve is the application of flocking control model. The black curve is the application of original cooperative obstacle avoidance model. The crimson curve is the improved cooperative obstacle avoidance model. Tanner's flocking control model based nodes group decelerates when it encounteres obstacle; efficiency of obstacle avoidance is very low. It did not reach the target after 100 seconds. Original cooperative obstacle avoidance model based nodes group has less waste of speed. Nodes group move along with the edge of obstacle and finally reach the target. However, obstacle and nodes group remained relatively static. It takes 90 seconds to reach the destination. Improved cooperative obstacle avoidance model took obstacle's velocity into consideration; it chose a better motion path. It takes only 60 seconds to reach the destination. The speed curve reveals that model keeps a high speed and efficiency to avoid the obstacle.

### 4.3. Simulation in Concave Obstacle Environment

**Figure 15:**
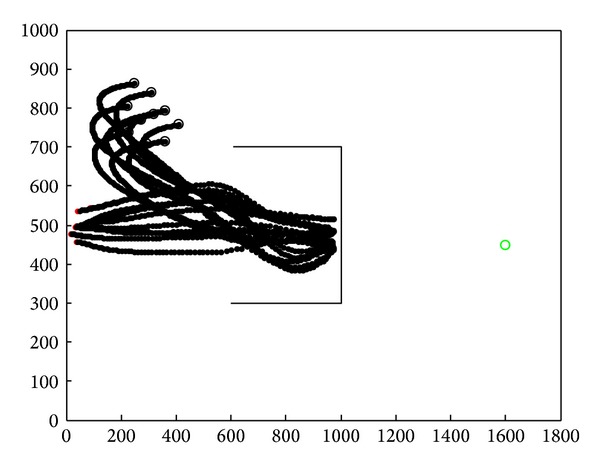
Concave obstacle avoidance by applying flocking control model.

**Figure 16:**
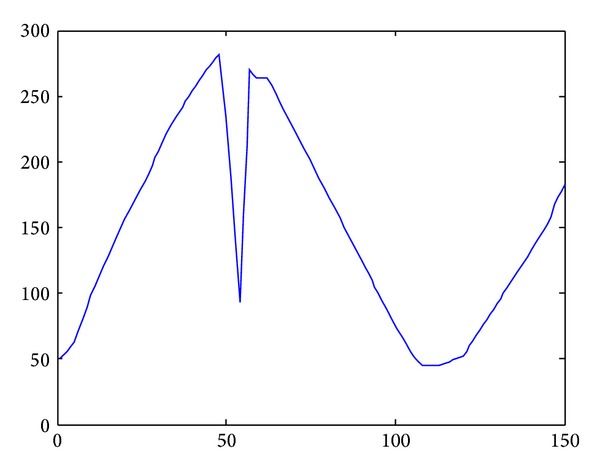
Speed curve by applying flocking control model.

In Figure 15, we applied flocking control model into concave obstacle environment. Flocking control model only exerts a repulsive force on nodes while nodes stay too close to the obstacle. After two decelerations in the concave obstacle, nodes group finally exit the concave obstacle. It takes a long procedure to make nodes adjust their velocity direction towards target again. What is more, nodes group might enter concave obstacle again, and the target tracking procedure fell into an infinite loop and cannot reach the target forever. The speed curve of the 10 nodes is shown in Figure 16. It takes 150 seconds for nodes group to reach the target.

**Figure 17:**
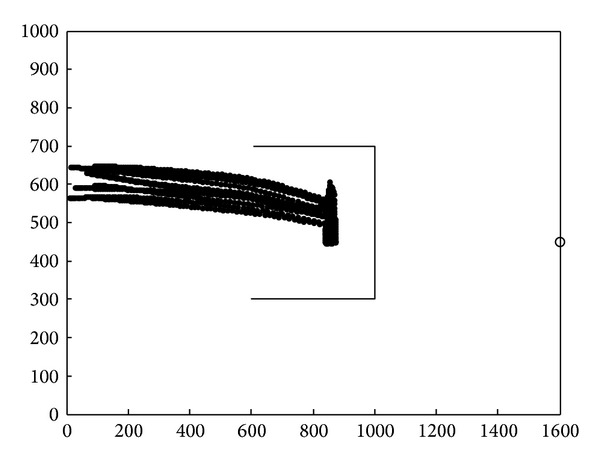
Concave obstacle avoidance by applying original cooperative obstacle avoidance model.

Applying original cooperative obstacle avoidance model into simulation, the result is shown in Figure 17.

**Figure 18:**
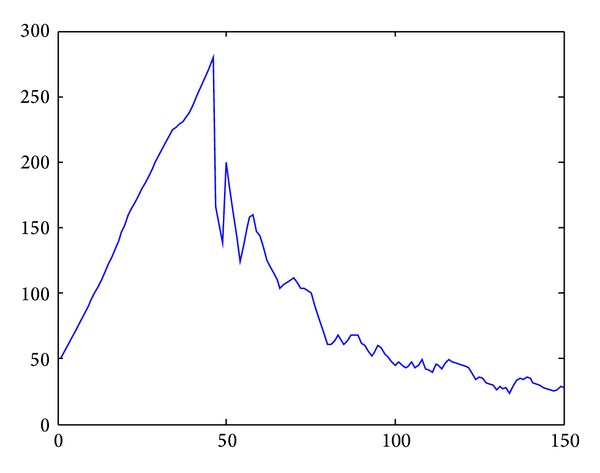
Speed curve by applying original cooperative obstacle avoidance model.

According to the SA algorithm, all the steering judgment is based on the target. Nodes group will make SA judgment for several times when it enteres the concave obstacle. But each judgment only let nodes group be trapped in the concave obstacle rather than exit. The average speed curve of 10 nodes is shown in Figure 18. Average speed of nodes group slowly decelerate but still could not get out of the concave obstacle. It did not reach the target after 150 seconds.

**Figure 19:**
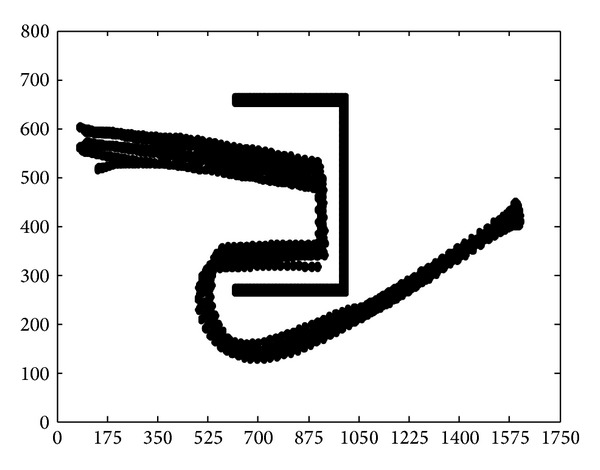
Concave obstacle avoidance by applying improved cooperative obstacle avoidance model.

**Figure 20:**
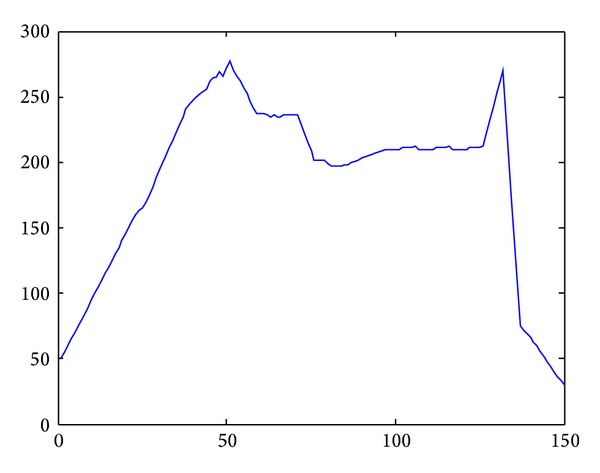
Speed curve by applying improved cooperative obstacle avoidance model.

Improved cooperative obstacle avoidance model takes concave obstacle into consideration; the application of this model is shown in Figure 19. When nodes group approach the obstacle at first time, improved model's SA judgment is the same as the original model. On second time, improved model chose a better path based on the local information. The gravitation force of obstacle let nodes group search the path along with the edge of obstacle and finally exited the concave obstacle. It takes 150 seconds to reach the destination. Average speed curve is shown in Figure 20.

**Figure 21:**
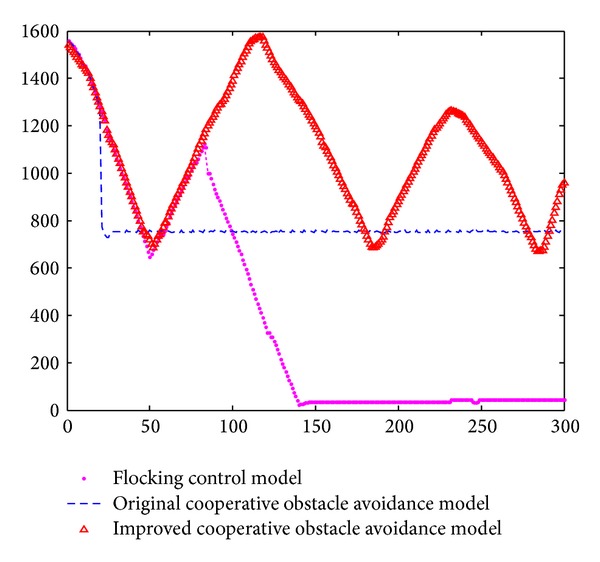
Average distance curve between nodes group and target.

The average distance curve between nodes and target is shown in Figure 21. The blue short-dotted curve is the application of flocking control model. Nodes group decelerated when it encountered obstacle; it took too much time for nodes group to adjust its velocity to move towards target. Nodes group reentered obstacle after exiting the concave obstacle. Thus, average distance between nodes and target rises and reduces alternately; nodes group cannot reach the target at the end. The blue solid curve is the application of original cooperative obstacle avoidance model. Based on the SA algorithm, nodes group made its SA steering judgment several times. However, each time of the SA steering judgment is always aimed at the target. Distance between nodes group and destination remains a constant value; nodes group did not reach the target too. The blue long-dotted curve is the application of improved cooperative obstacle avoidance model. After two SA judgments, nodes could already determine that it was in the concave obstacle. Although the procedure of nodes group search path along with edge of obstacle temporary canceled the gravitation force of target, the velocity of nodes group was adjusted very quick, and nodes group reaches the target at last.

In conclusion, the cooperative obstacle avoidance model established in this paper has a considerable improvement in both efficiency and stability in obstacle avoidance. What is more, improvement of the model based on complex environment lets model have better flexibility. Cooperative obstacle avoidance model lets nodes group move towards the target cooperatively and avoids the complex obstacles in the environment.

## 5. Conclusion

This paper studied the features of target tracking in mobile wireless sensor network and the concept, features, category, application areas of flocking control mode, and obstacle avoidance algorithm. This paper introduced the flocking control model which was realized by Tanner et al., combined with the improved SA algorithm and introduced cooperative obstacle avoidance model. Aimed at the defects of the model in complex obstacle environment, nodes group could predict the motion tendency of the obstacle, so that nodes could make a better steering judgment. While nodes group encountered concave obstacles, improved model lets nodes group search the path along with the edge of obstacle and cancels the gravitation force of the target. It restores the gravitation force after nodes group exits the concave obstacle. Simulation results demonstrated the efficiency and the stability of the model in complex obstacle environment.
